# Phenotypic Characterization of Peripheral T Cells and Their Dynamics in Scrub Typhus Patients

**DOI:** 10.1371/journal.pntd.0001789

**Published:** 2012-08-14

**Authors:** Bon-A Cho, Youngho Ko, Yeon-Sook Kim, Sanguk Kim, Myung-Sik Choi, Ik-Sang Kim, Hang-Rae Kim, Nam-Hyuk Cho

**Affiliations:** 1 Department of Microbiology and Immunology, Seoul National University College of Medicine, Seoul, Republic of Korea; 2 Department of Anatomy, Seoul National University College of Medicine, Seoul, Republic of Korea; 3 Institute of Endemic Disease, Seoul National University Medical Research Center, Seoul, Republic of Korea; 4 Divisions of Infectious Diseases, Chungnam National University Hospital, Daejeon, Republic of Korea; 5 Department of Life Science and School of Interdisciplinary Bioscience and Bioengineering, Pohang University of Science and Technology, Pohang, Kyungbuk, Republic of Korea; 6 Bundang Hospital, Seoul, Republic of Korea; University of Texas Medical Branch, United States of America

## Abstract

**Background:**

Scrub typhus, caused by *Orientia tsutsugamushi* infection, is one of the main causes of febrile illness in the Asia-Pacific region. Although cell-mediated immunity plays an important role in protection, little is known about the phenotypic changes and dynamics of leukocytes in scrub typhus patients.

**Methodology/Principal Findings:**

To reveal the underlying mechanisms of immunological pathogenesis, we extensively analyzed peripheral blood leukocytes, especially T cells, during acute and convalescent phases of infection in human patients and compared with healthy volunteers. We observed neutrophilia and CD4^+^ T lymphopenia in the acute phase of infection, followed by proliferation of CD8^+^ T cells during the convalescent phase. Massive T cell apoptosis was detected in the acute phase and preferential increase of CD8^+^ T cells with activated phenotypes was observed in both acute and convalescent phases, which might be associated or correlated with elevated serum IL-7 and IL-15. Interestingly, peripheral Treg cells were significantly down-regulated throughout the disease course.

**Conclusions/Significance:**

The remarkable decrease of CD4^+^ T cells, including Treg cells, during the acute phase of infection may contribute to the loss of immunological memory that are often observed in vaccine studies and recurrent human infection.

## Author Summary

Scrub typhus is an acute febrile illness caused by *Orientia tsutsugamushi* infection. It has been estimated that one billion people are at risk and one million new cases arise each year in Asian-pacific region. Despite of aggressive attempts to develop a prophylactic vaccine against scrub typhus during the last several decades, all approaches have failed to generate long lasting immunity. In addition, little is known about the immunological pathogenesis of scrub typhus. To understand the pathogenic mechanisms of this infectious disease, we extensively analyzed peripheral leukocytes, especially T cells, in Korean scrub typhus patients and compared with healthy volunteers. We observed neutrophilia and CD4^+^/T lymphopenia in the acute phase of infection, followed by proliferation of CD8^+^ T cells during the convalescent phase. Massive T cell apoptosis was detected in the acute phase and a preferential increase of CD8^+^ T cells with activated phenotypes was observed in both acute and convalescent phases. The remarkable decrease of CD4^+^ T cells, including Treg cells, during the acute phase of infection may contribute to the loss of immunological memory and generate helpless but unregulated cytotoxic T cell responses observed in vaccine studies and recurrent human infection.

## Introduction

Scrub typhus is an acute febrile illness caused by *Orientia tsutsugamushi* infection, an obligate intracellular bacterium, following the bite of infected larval mites [1]. While the disease is confined geographically to the Asia-Pacific region, it has been estimated that one billion people are at risk and one million new cases arise each year in the endemic region [2]. In addition, this infectious disease has recently become an important public health issue due to regional outbreaks [3], [4] and new emergence [5], [6].

Clinical presentations of scrub typhus, typically characterized by eschar, fever, rash, lymphadenopathy, and myalgia, can vary in severity from a mild and self-limiting flu-like syndrome to a life-threatening disease [1], [7]. If not properly treated in the early stage or left untreated, patients often develop severe pneumonitis, meningitis, renal failure, myocarditis, and disseminated intravascular coagulation [7], [8]. The diverse pathologic changes in multiple organs are mainly due to focal or disseminated multi-organ vasculitis or perivasculitis of small blood vessels since *O. tsutsugamushi* primarily infects endothelial cells [9], [10], [11]. Although it has been suggested that adaptive immune cells, such as CD8^+^ T cells, may cause injury to vascular endothelial cells, leading to vasculitis or perivascularitis during infections by diverse intracellular pathogens [12], [13], little is known about the underlying mechanisms of the pathologic damage observed in scrub typhus patients.

Despite aggressive attempts to develop a prophylactic vaccine against scrub typhus, all approaches have failed to generate long lasting immunity in humans [6]. It has been well established that effective cell-mediated immunity is required for protection against *Orientia* infection in murine models [1], [6]. Mice infected with *O. tsutsugamushi* show enhanced IFN-γ expression [14], [15] and transfer of IFN-γ-producing Th1 cells protects mice against *O. tsutsugamushi* [16]. In a nonhuman primate model, *Orientia* infection or vaccination induces antigen-specific proliferation of lymphocytes and IFN-γ production by peripheral blood mononuclear cells (PBMCs) [17], [18]. However, long-lasting memory responses have never been achieved in primate models [18]. It is also notable that rapid immunosuppression at both humoral and cellular levels has been consistently observed in immunized animals right after bacterial challenge [17], [19] and recurrent human infection is relatively common in highly endemic regions despite T cell activation during primary infection [20].

Here, we examined phenotypic characteristics of peripheral blood leukocytes and their dynamics in scrub typhus patients. Comparative analysis of scrub typhus patients' immune cells during acute and convalescent phases of infection with those from healthy controls revealed a dynamic fluctuation of leukocyte populations, especially T cells, during the course of the disease.

## Materials and Methods

### Ethics statements

Ethical approval for this work was granted by the Institutional Review Board of both Seoul National University Hospital (IRB No. 0-1001-039-307) and Chungnam National University Hospital (IRB No. 2008-10-08). All patients and healthy volunteers provided written informed consent prior to sample collection.

### Patient samples

Human peripheral blood was drawn from healthy volunteers (n = 9) and scrub typhus patients (n = 34) after obtaining informed consent at Chungnam National University Hopspital in Deajon, South Korea. Scrub typhus was confirmed by the presence of *O. tsutsugamushi*-specific antibody titer greater than 1∶400 in serum from patients with acute febrile disease and/or at least a four-fold increase in antibody titer. Scrub typhus patients and healthy controls were matched for gender (*p* = 0.455, Fisher's exact test) and age (age mean ± SD, patients 57.6±13.8 *versus* healthy 63.0±3.3 years, Student's *t* test, *p* = 0.2687). Blood samples were collected from each patient at two time points, once during the acute phase (samples drawn upon admission and before antibiotic treatment) and again during the convalescent phase (samples drawn after antibiotic treatment) of scrub typhus. Acute phase is defined as the period between the onset of symptoms to admission (Median = 8 days after onset of symptoms, 95% confidence interval (CI) = 6.4 to 14.3, range 1 to 30 days). Convalescent phase begins about 10 days after the acute phase (Median = 19.5 days after onset of symptoms, 95% confidence interval (CI) = 18.0 to 26.0, range 13 to 43days). Among the patients, four have diabetes and three have been diagnosed with cancer. All of them had been properly treated before the *Orientia* infection, showed no prior signs of immunodeficiency in their medical records, and successfully recovered from scrub typhus after antibiotics therapy. The individual clinical information of all the scrub typhus patients enrolled in the study are presented in Table S1 and summarized in Table S2.

### Flow cytometric analysis

The leukocytes differential count was determined using a Sysmex XE-2100 hematology analyzer (Sysmex Corporation, Kobe, Japan), which differentiates leukocytes by simultaneously measuring volume, structure, and fluorescence [21]. PBMCs were isolated by standard density centrifugation with Histopaque (GE Healthcare, Little Chalfont, United Kingdom) and stored at liquid nitrogen after suspension in freezing media (50% fetal bovine serum, 10% DMSO, and 40% RPMI-1640, Invitrogen, Carlsbad, CA) until analysis. Each subset of leukocytes was analyzed simultaneously in order to minimize the variation due to staining procedures. Cells were stained with various antibodies to analyze the frequency of leukocyte populations and their cellular characteristics as follows; to analyze the frequencies and absolute counts of CD4^+^ and CD8^+^ T cell subsets, cells were stained with antibodies against CD3 and CD8. The frequency of each population was examined after gating lymphocyte population (1). The frequencies and phenotypic characteristics of CD4^+^ and CD8^+^ T cell subsets were further analyzed by co-staining with antibodies to surface antigens, CD4 and CD8 in addition to CD25, CD45RA, CCR4, CCR7, CXCR3, Fas (CD95), PD-1 (all from BD Biosciences, San Jose, CA), IL-7Rα(CD127) (R&D systems, Minneapolis, MN)or isotype controls. Some cells were co-stained with annexin V (BD Biosciences) in order to measure the cellular apoptosis. For staining intracellular antigens, cells were stained with anti-Foxp3 (Biolegend, San Diego, CA), CTLA-4, Ki-67 (BD Biosciences) antibodies, or isotype controls after fixation and permeabilization. For measuring the frequencies of natural killer (NK) cells, PBMCs were stained with antibodies to CD3, CD4, CD8, and CD56 (BD Biosciences). Representative gating strategies for each assay are presented in Figure S1, S2, S3. Samples (2×10^5^∼1×10^6^ events per sample) were collected and analyzed with an LSRII® (BD Immunocytometry Systems, San Jose, CA). Data were analyzed using FlowJo® software (Tree Star, Ashland, OR). The absolute count of each lymphocyte population in the patients were calculated based on leukocyte differential counts and frequency data obtained from FACS analysis are presented in Table S3 and summarized in Table S4.

### Multiplex cytokine assays

Bio-plex cytokine assay (Bio-Rad Inc., Hercules, CA) was used to quantify soluble IL-7, IL-10, IL-15, and IFN-γ in the serum according to the manufacturer's instructions. Samples were measured and analyzed on a Bio-Plex 200 system (Bio-Rad) in combination with the Bio-Plex Manager software (Bio-Rad).

### Statistical analysis

All data are expressed as mean ± standard deviation or mean ± standard error of the mean. The two-tailed Student's *t* test, Wilcoxon sign-rank test, and Mann-Whitney *U* test were used to compare measurable variables between patients and healthy controls, or patients in acute phase and convalescent phase of infection. *p* values<0.05 were considered statistically significant. All statistical analyses were accomplished using GraphPad Prism 5.01 (GraphPad Software Inc., La Jolla, CA).

## Results

### Alteration of leukocyte populations in peripheral blood of scrub typhus patients

**Figure 1:**
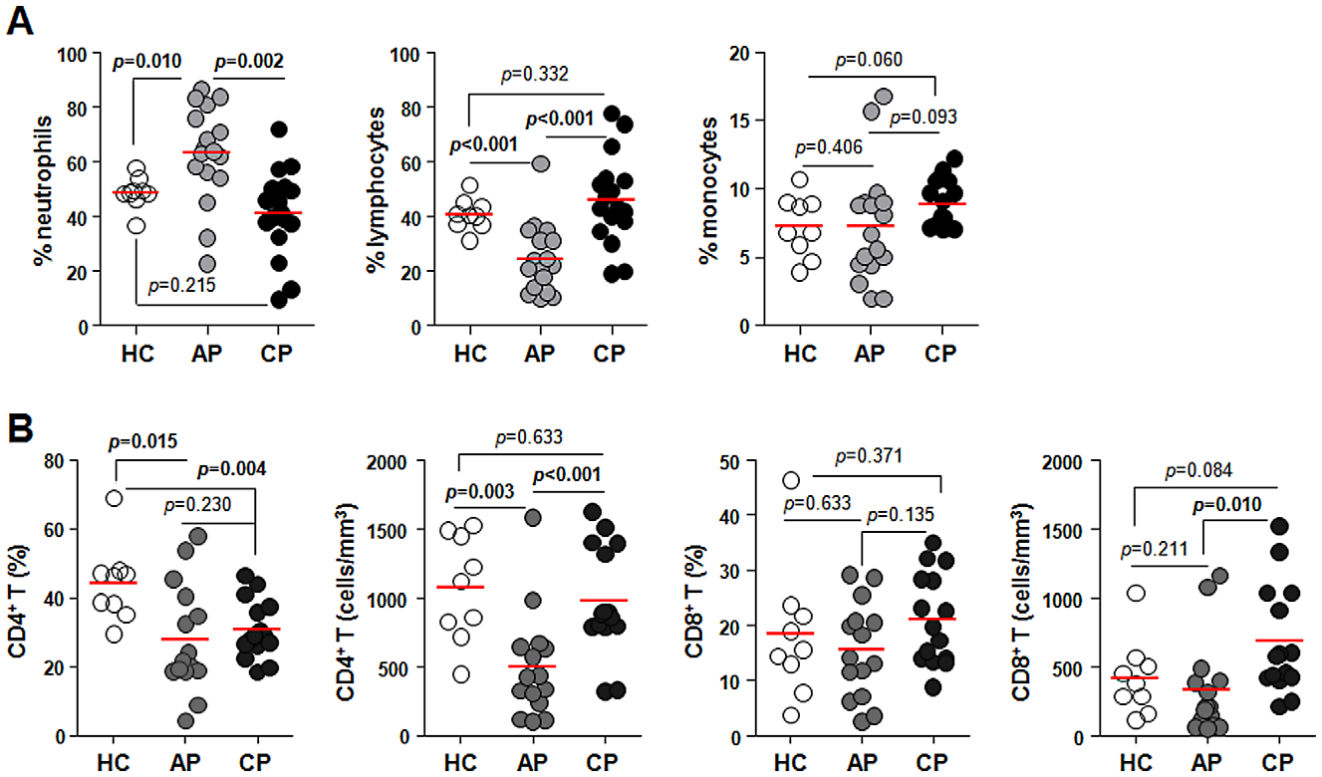
Profiles of peripheral blood leukocytes and T lymphocytes in scrub typhus patients. A. The frequencies (%) of neutrophils, lymphocytes, and monocytes in blood leukocytes from healthy controls (HC, n = 9, open circle) were determined using a hematology analyzer and compared to frequencies in scrub typhus patients during acute phase (AP, n = 17, gray circle) or convalescent phase (CP, n = 17, black circle) of infection. B. Peripheral blood mononuclear cells (PBMCs) from scrub typhus patients and healthy controls were stained with anti-CD3 and CD8 antibodies and analyzed by flow cytometry (see Figure S1). The frequencies (% in lymphocytes) and absolute numbers (cells/mm^2^) of CD4^+^ or CD8^+^ T cells in PBMCs of the patients (AP, n = 15 and CP, n = 15) and healthy controls (HC, n = 9) were compared. The absolute number of each leukocyte subset was calculated based on the leukocyte differential counts and the frequency information obtained from flow cytometry. For example, CD4^+^ T cells count = (lymphocyte count) x (% of CD4+ T cells)/100. The data of each individual person are presented in Table S3. Red bars indicate the mean value and *p* values were obtained using the Mann-Whitney *U* test or Wilcoxon signed-rank test. Statistically significant *p* values (<0.05) are shown in bold.

Whole blood leukocytes were collected from scrub typhus patients during acute and convalescent phases and analyzed for changes in frequencies and subsets. The absolute numbers (cells/mm^3^, mean ± SD) of the peripheral blood leukocytes were generally increased in the patients at both acute (7,175±2,296) and convalescent (7,012±1,844) phases when compared to those of healthy controls (5,794±742). Significant changes were detected in neutrophil and lymphocyte populations (Figure 1A). The frequency (%, mean ± SD) of neutrophils was significantly increased during acute phase (62.96±17.66) and then returned to baseline levels during convalescent phase (41.28±15.72; healthy controls, 48.61±5.70). In contrast, lymphocytes were significantly reduced (24.49±12.73) during acute phase and returned to baseline (45.91±16.23) in convalescent phase compared to healthy controls (40.71±5.62). The absolute numbers of each leukocyte population were also changed in similar pattern (Table S3 and S4). To examine the population changes of lymphocytes in detail, PBMCs were further analyzed by flow cytometry (Figure 1B). The decrease of lymphocytes in the patients was mainly due to a significant reduction in CD4^+^ T cells in both frequency (%, 27.96±15.70) and number (cells/mm^3^, 498.5±387.9) during acute phase compared with healthy controls (%, 44.33±11.24; cells/mm^3^, 1,072.0±382.4). CD8^+^ T cells were not significantly different in frequency during acute and convalescent phase but were significantly increased in total numbers during the convalescent phase (681.8±395.4) when compared to that of acute phase (329.4±349.5).

### Apoptosis and proliferation of T cells during *Orientia* infection

**Figure 2:**
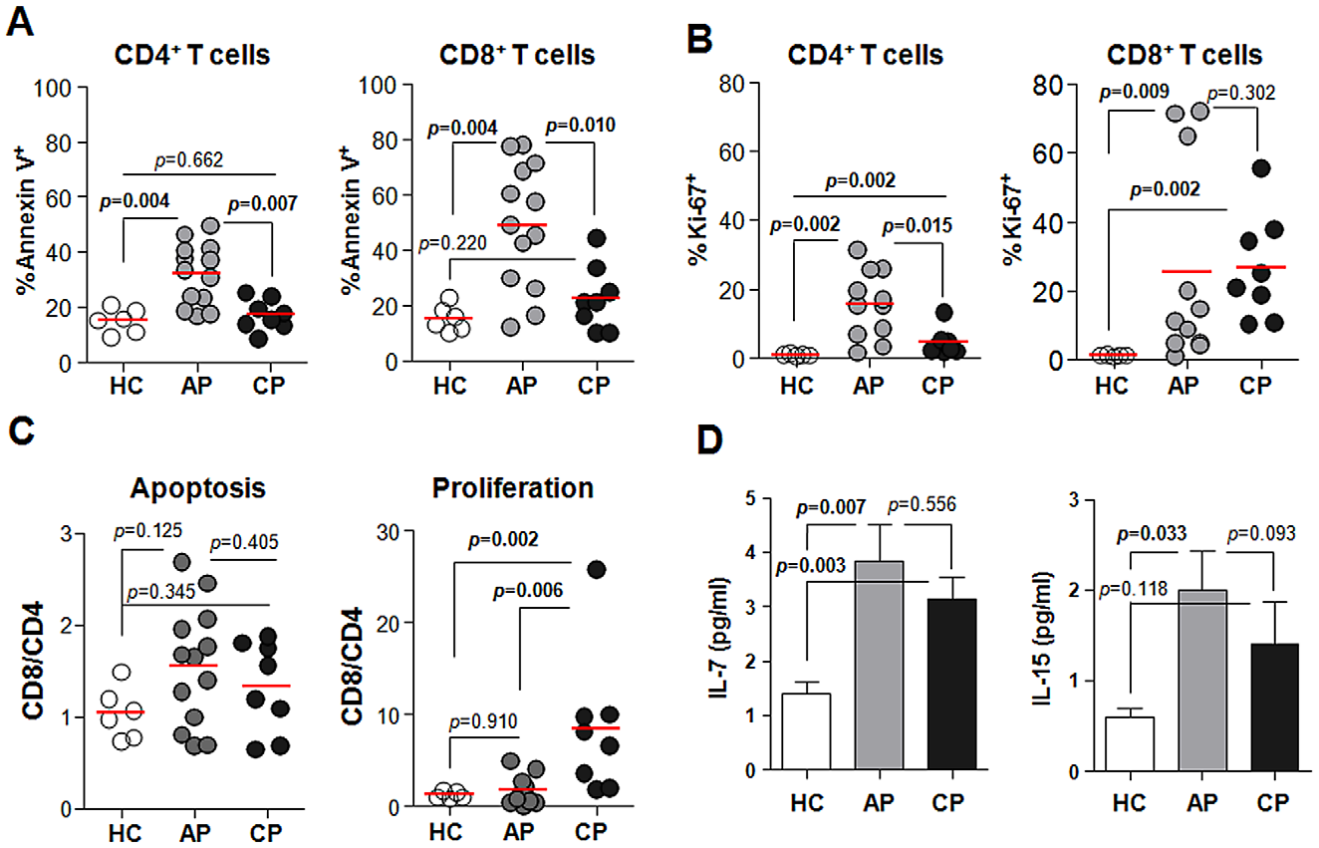
Apoptosis and proliferation of T cells during *Orientia* infection. A and B. PBMCs were stained with antibodies against CD4, CD8, Annexin V, or Ki-67 and then analyzed by flow cytometry. The frequencies of Annexin V- (*A*) or Ki-67- (*B*) positive cells in CD4^+^ and CD8^+^ T cells were compared among healthy controls (HC, n = 6, open circle) and scrub typhus patients at acute phase (AP, n = 13–15, gray circle) or convalescent phase (CP, n = 6, black circle). Red bars indicate the mean value. C. The CD8/CD4 ratio of apoptotic or proliferating cells were compared among the patients and healthy volunteers. D. The levels of IL-7 and IL-15 (pg/ml) in the sera were measured and compared. Error bars indicate standard error from the mean value (C *and* D). *p* values were obtained using the Mann-Whitney *U* test. Statistically significant *p* values (<0.05) are shown in bold.

In order to investigate the mechanism of reduced T cell frequencies during *Orientia* infection, we next determined the frequencies of apoptotic and proliferating T cells in the patients' blood by flow cytometry. Apoptotic T cells were identified by staining with annexin V and proliferating cells were evaluated by a cellular proliferation marker, Ki-67^+^ [22]. As shown in Figure 2A, the frequencies of apoptotic CD4^+^ (%, 29.60±12.58) and CD8^+^ (43.87±24.97) T cells within each T cell subset were significantly increased during acute phase of infection when compared with healthy controls (CD4^+^, 15.23±4.39; CD8^+^, 15.32±4.68). During convalescent phase, the percentage of apoptotic CD4^+^ T cells returned to baseline levels but the percentage of apoptotic CD8^+^ T cells (27.00±10.36) was still higher than in healthy controls. Cellular proliferation was also significantly increased during acute phase of infection in both CD4^+^ and CD8^+^ populations (Figure 2B). During convalescent phase, however, the frequency of proliferating CD8^+^ T cells (32.18±13.65) was remarkably increased when compared with healthy controls (1.13±0.59), whereas proliferation of CD4^+^ T cells returned to basal level. These results indicate that both CD4^+^ and CD8^+^ T cells undergo rapid turnover during acute phase and CD8^+^ T cells proliferate more actively than CD4^+^ T cells during convalescent phase (Figure 2C). To investigate the potential mechanisms of the rapid turnover and proliferation of T cells in scrub typhus patients, we measured the amount of soluble IL-7 and IL-15 in the serum of patients since these cytokines induce the proliferation of and enhance the survival of T cells in mice and humans [23]. The levels of IL-7 were significantly higher in the patients' sera (pg/ml, 4.12±2.34 and 3.14±1.58 in acute and convalescent phase respectively) than in healthy controls (1.40±0.70) (Figure 2D). The levels of soluble IL-15 in infected patients were also increased during acute phase (2.00±1.93) when compared with healthy controls (0.61±0.30).

### Changes in type 1 and type 2 effector T cells during *Orientia* infection

**Figure 3:**
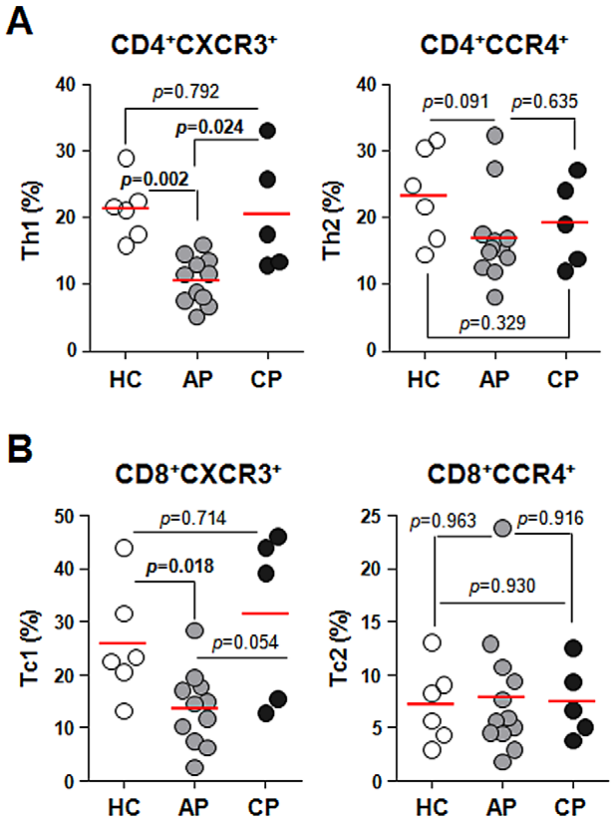
Profiles of type 1 and type 2 T cells in peripheral blood of scrub typhus patients. PBMCs were stained with antibodies against CXCR3, CCR4, CD4, and CD8 and then analyzed on a flow cytometer. A. The frequencies of Th1 (CD4^+^CXCR3^+^) or Th2 (CD4^+^CCR4^+^) within CD4^+^ T cells were compared among healthy controls (HC, n = 6, open circle) and the patients in acute phase (AP, n = 12, gray circle) or convalescent phase (CP, n = 5, black circle). B. The frequencies of Tc1 (CD8^+^CXCR3^+^) and Tc2 (CD8^+^CCR4^+^) within CD8^+^ T cells were compared among healthy controls and the patients as in *A*. Red bars indicate the mean value and *p* values were obtained using the Mann-Whitney *U* test. Statistically significant *p* values (<0.05) are shown in bold.

Since the balance of effector T cells plays a critical role in controlling an infection, we next examined the overall changes in effector phenotypes of T cells in the patients. Human effector T cells can be categorized into type 1 CD4^+^ (Th1) and CD8^+^ (Tc1) T cells or type 2 CD4^+^ (Th2) and CD8^+^ (Tc2) T cells, based on preferential expression of CXCR3 or CCR4, respectively [24], [25]. As shown in Figure 3, the frequencies of CD4^+^CXCR3^+^ Th1 (%, 10.59±3.53 in acute phase *versus* 21.28±4.60 in healthy controls) and CD8^+^CXCR3^+^ Tc1 cells (13.44±6.82 *versus* 25.77±10.66) were transiently reduced in infected patients, whereas CD4^+^CCR4^+^ Th2 and CD8^+^CCR4^+^ Tc2 cells were not changed significantly.

### Reduction of CD4^+^ regulatory T cells during *Orientia* infection

**Figure 4:**
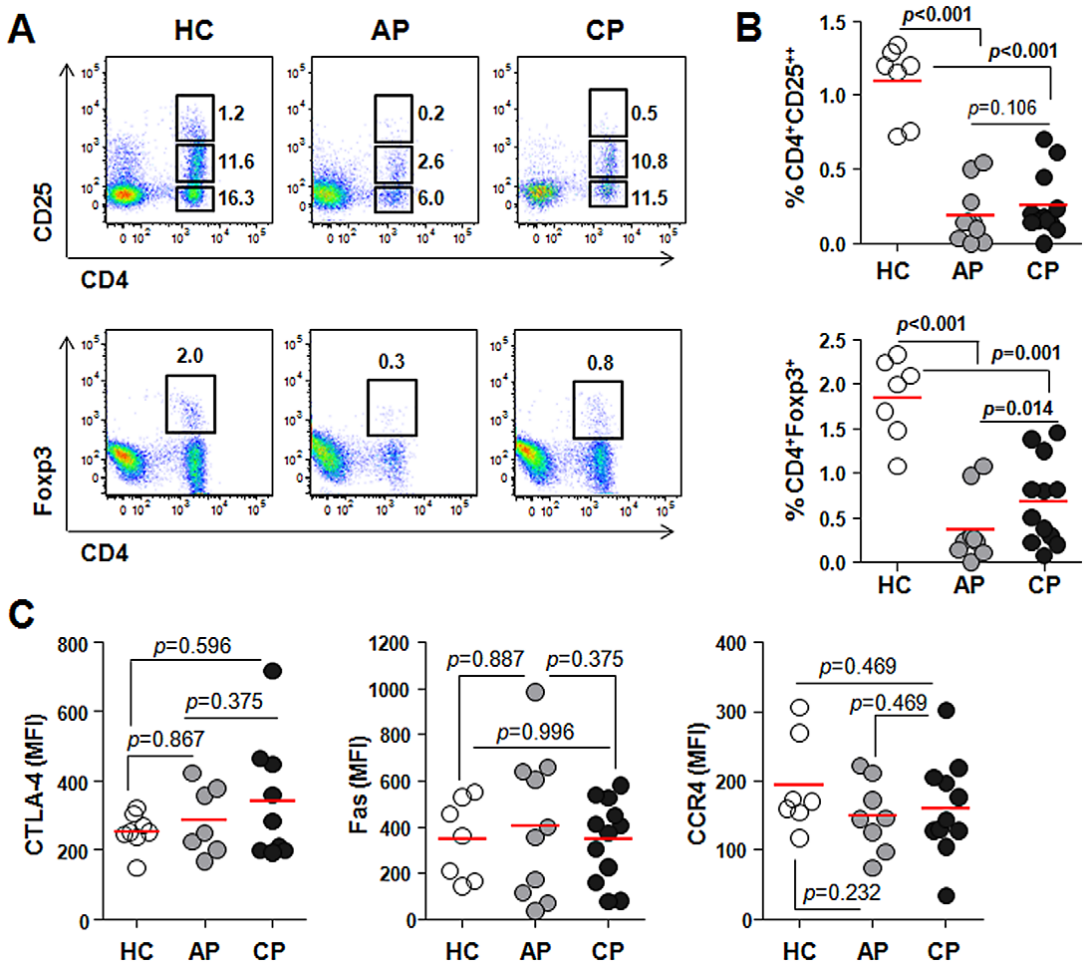
Profiles of CD4^+^Foxp3^+^ or CD4^+^CD25^++^ regulatory T cells in scrub typhus patients. A. PBMCs were stained with antibodies against CD4 and CD25 or Foxp3 and then analyzed on a flow cytometer. Representative dot plots show the identification of CD25^++^ T cells (upper panels) or Foxp3^+^ T cells (lower panels) within CD4^+^ T cells. Numbers in the plots indicate the frequencies (%) of the gated cells in total PBMCs. B. The frequency of CD4^+^CD25^++^ or CD4^+^Foxp3^+^ T cells were compared between healthy controls (HC, n = 7, open circle) and scrub typhus patients at acute phase (AP, n = 10, gray circle) or convalescent phase (CP, n = 12, black circle). C. PBMCs were stained with antibodies against CD4 and Fas or CCR4, followed by intracellular staining of Foxp3 and/or CTLA-4 after fixation and permeablization. Mean fluorescent Intensities (MFI) representing CTLA-4, Fas, or CCR4 expression in CD4^+^Foxp3^+^ regulatory cells from healthy controls (HC, n = 7–8, open circle) or the patients (AP, n = 7–10, gray circle and CP, n = 9–12, black circle) were compared. Red bars indicate the mean value and *p* values were obtained using the Mann-Whitney *U* test or Wilcoxon signed-rank test. Statistically significant *p* values (<0.05) are shown in bold.

Regulatory T cells (Treg) expressing Foxp3 and high levels of CD25 (CD25^++^) are required for maintaining peripheral tolerance to self-antigen and controlling immune responses during an infection by inhibiting the activation of effector T cells [26]. The frequency of Treg cells and their phenotypical characteristics in the peripheral blood of scrub typhus patients were examined for the first time. Foxp3 expression correlated well with the high level of CD25 surface expression in CD4^+^ T cells (Figure S4). Interestingly, both CD4^+^CD25^++^ and CD4^+^Foxp3^+^ T cells were significantly reduced in scrub typhus patients (CD4^+^CD25^++^, %, 0.191±0.194 in acute phase and 0.256±0.22 in convalescent phase; CD4^+^Foxp3^+^, 0.363±0.361 in acute phase and 0.683±0.480 in convalescent phase) when compared with healthy controls (CD4^+^CD25^++^, 1.096±0.250; CD4^+^Foxp3^+^, 1.844±0.451) (Figure 4A and B). Treg cells were generally increased during convalescent phase compared to that of acute phase, but were still significantly lower than the levels in healthy controls. To further investigate whether the cellular phenotypes of Treg cells are altered during *Orientia* infection, we determined the expression levels of CTLA-4, Fas, and CCR4, which are involved in suppressor function and migration [26], [27]. Despite the significant reduction in Treg frequency in the patients' blood, the expression levels of CTLA-4, Fas, and CCR4 in Treg cells were quite similar to those of healthy controls (Figure 4C).

### Changes in effector and memory T cell subsets during *Orientia* infection

**Figure 5:**
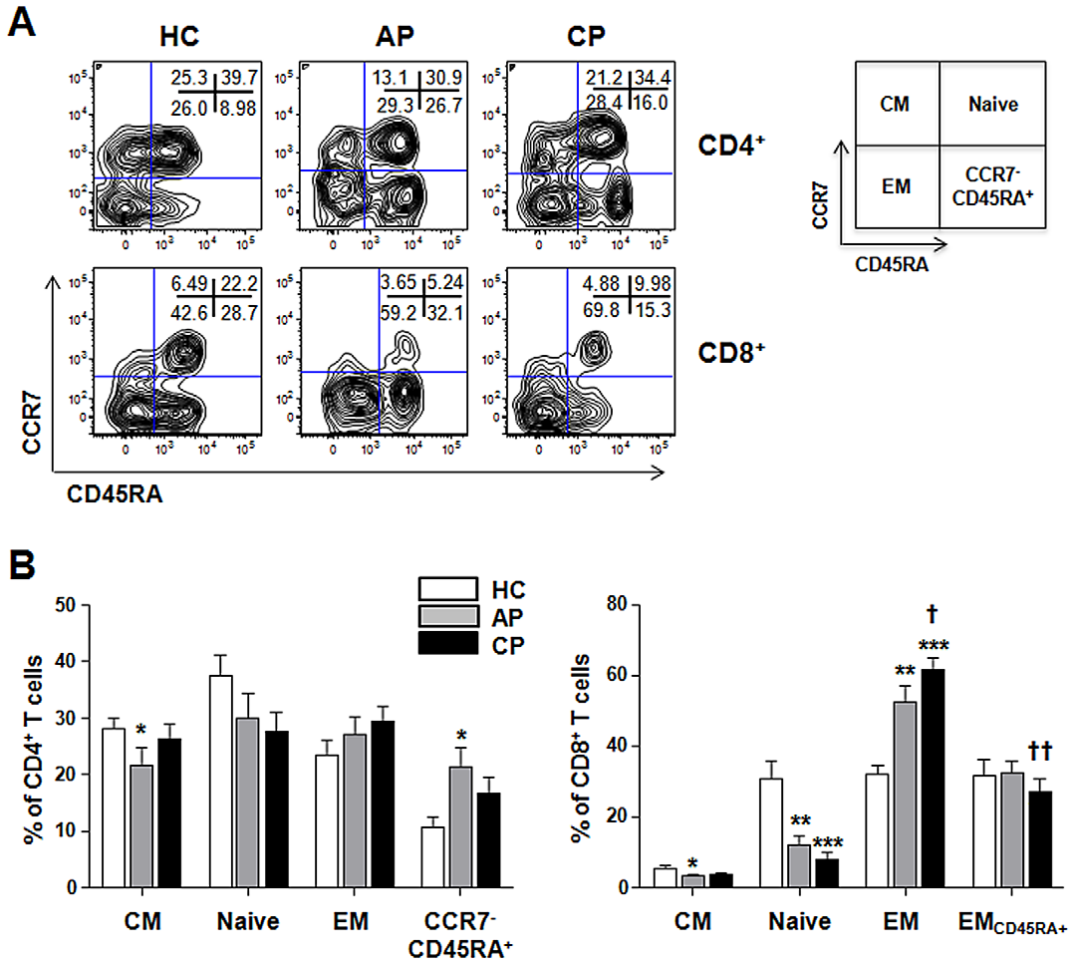
Changes of effector and memory T cell subsets in scrub typhus patients. A. Representative contour plots show the frequencies of each subsets within CD4^+^ (upper panels) or CD8^+^ (lower panels) T cell populations. Numbers within the plots indicate the percentage of each subset. B. The frequencies of naïve (CCR7^+^CD45RA^+^), central memory (CM, CCR7^+^CD45RA^−^), and effector memory (EM, CCR7^−^CD45RA^−^), CD45RA^+^ EM (EM_CD45RA+_, for CD8^+^ T cells), or CCR7^−^CD45RA^+^ (for CD4^+^ T cells) T-cell subsets in healthy controls (HC, n = 9) and scrub typhus patients at acute phase (AP, n = 15) or convalescent phase (CP, n = 17) were compared. Error bars indicate standard error of mean values. *p* values were obtained using the Mann-Whitney *U* test or Wilcoxon signed-rank test. *, *p*<0.05; **, *p*<0.01; and ***, *p*<0.001 (compared with that of healthy control); †, *p*<0.05 and ††, *p*<0.01 (compared with that of the patients at acute phase).

Next, we investigated the changes in the distribution of effector and memory T cell subsets in the peripheral blood of scrub typhus patients. The differentiation status of T cells was defined based on differential staining of CCR7 and CD45RA (Figure 5) [28]. Based on phenotypic markers, T cell subsets can be classified into 4 four subsets: naïve (CCR7^+^CD45RA^+^), central memory (CM, CCR7^+^CD45RA^−^), effector memory (EM, CCR7^−^CD45RA^−^), and CCR7^−^CD45RA^+^ cells (EM_CD45RA+_ for CD8^+^ and CCR7^−^CD45RA^+^ for CD4^+^ T cells). In contrast to the significant reduction of CD4^+^ T cells during acute phase of infection (Figure 1), the distribution of CD4^+^ T cell subsets in healthy controls and scrub typhus patients were generally consistent, with the exception of CM (%, 21.7±12.6 in acute phase *versus* 28.0±6.8 in healthy controls) and CCR7^−^CD45RA^+^ (21.3±13.1 *versus* 9.1±5.8) (Figure 5A and B). For CD8^+^ T cells, the naive subset was significantly decreased in scrub typhus patients (11.9±10.5 in acute and 8.0±7.8 in convalescent phase) compared to that of healthy controls (29.4±16.2). In contrast, the frequency of EM CD8^+^T cells in the patients (52.6±17.4 in acute and 61.4±15.2 in convalescent phase) was significantly increased (35.1±9.7 in healthy controls). The frequency of EM CD8^+^ T cells was further increased during the convalescent phase compared to the acute phase (*p* = 0.048), whereas EM_CD45RA+_ CD8^+^ T cells were significantly decreased during the convalescent phase (*p* = 0.008).

**Figure 6:**
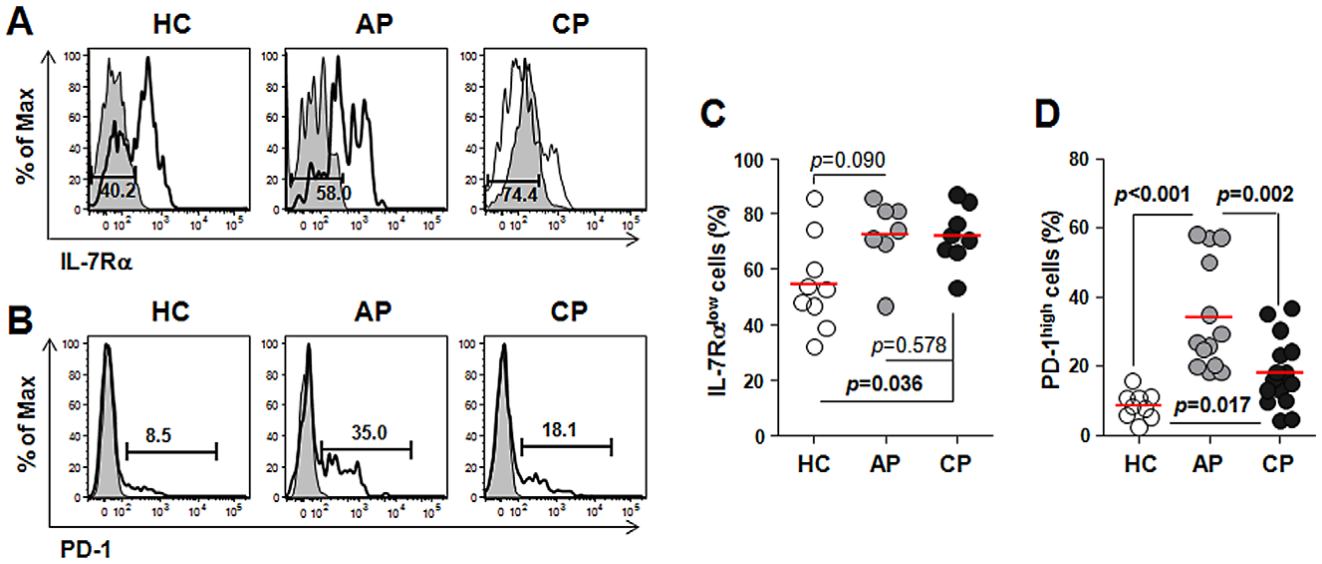
Analysis of activated CD8^+^ T cells in peripheral bloods of scrub typhus patients. PBMCs were stained with antibodies against CD8 and PD-1 or IL-7Rα and then analyzed on a flow cytometer. A and B. Representative histograms show the frequencies of IL-7Rα^low^ cells (A) and PD-1^high^ cells (B) within CD8^+^ T cells. Gray histogram, isotype control. C. The frequency of IL-7Rα^low^CD8^+^ T cells from healthy controls (HC, n = 9, open circle) is compared with that of scrub typhus patients at acute phase (AP, n = 7, gray circle) or convalescent phase (CP, n = 8, black circle). D. The frequencies of PD-1^high^CD8^+^ T cells were compared among healthy controls (n = 9) and the patients (AP, n = 13; CP, n = 15). Red bars indicate the mean value and *p* values were obtained using the Mann-Whitney *U* test or Wilcoxon signed-rank test. Statistically significant *p* values (<0.05) are shown in bold.

The dramatic increase in EM CD8^+^ T cells in scrub typhus patients prompted us to further investigate the activation status of CD8^+^ T cells in patients. We evaluated the distribution of CD8^+^ T cells expressing low levels of IL-7 receptor alpha (IL-7Rα^low^) or high levels of programmed death-1 (PD-1^high^) within the CD8^+^ T cell population (Figure 6). These subpopulations mainly include antigen-experienced EM subsets and activated CD8^+^ T cells [29], [30]. As shown in Figure 6, the frequencies of IL-7Rα^low^ subsets and PD-1^high^ CD8^+^ T cells were generally increased in scrub typhus patients. While IL-7Rα^low^ CD8^+^ T cells comprised more than 70% of the total CD8^+^ T cells throughout the infection, PD-1^high^ cells did not persist and were reduced during the convalescent phase. Taken together, these data suggest that the major alterations in CD8^+^ T cell populations in scrub typhus patients might be due to the proliferation of activated CD8^+^ T cells that differentiated from T cells in early stages (naïve and CM T cells).

## Discussion

Like other infectious diseases, *Orientia* infection generally induces marked increases in total blood leukocytes. Since all the patients were treated with antibiotics, this may affect the immune responses seen in the convalescent phase. Nevertheless, neutrophilia is dominant in the acute phase and is followed by lymphocytosis, largely due to a rise in CD8^+^ T cells, during the convalescent phase in scrub typhus patients, as previously reported [20], [31]. During acute phase, however, we observed a significant reduction in T cell frequencies and total numbers, especially CD4^+^ cells (Figure 1B). Acute CD4^+^ T-lymphopenia has also been observed in other infectious diseases [32], [33], [34] and might be explained by the migration of circulating lymphocytes to inflamed tissues or by apoptotic cell death [35]. Here, we detected a significant increase in apoptosis in peripheral T cells during acute phase (Figure 2A) as observed previously in a mouse model infected with *O. tsutsugamushi* [36]. One mechanism for T cell apoptosis is activation-induced cell death (AICD), which occurs after the expansion of T cells responding to antigenic stimulation and increased IL-2 [37]. The increase of apoptotic cell death in T cells observed in scrub typhus patients, however, may not solely be due to AICD since it has been shown that the serum IL-2 levels in scrub typhus patients do not change during the symptomatic period [38]. In addition, the massive apoptosis of peripheral T cells observed in this study (Figure 2A), is quite similar to the sepsis phenotype [35] in which engagement of TCRs by cognate antigen is not required for T cell apoptosis [39]. Although the underlying mechanisms of T cell apoptosis in scrub typhus patients needs to be defined, the massive apoptotic death of T cells might contribute to the transient immunosuppression observed in animals experimentally challenged with *O. tsutsugamushi* [17], [19]. Previously, it was also reported that antigen-specific CD4^+^ T cells are rapidly deleted from blood after infection with several pathogens such as *Anaplasma marginales* [40], *Plasmodium* [41], and *Brugia Pahangi* [42]. Rapid decline of antigen-specific CD4^+^ T cells during the acute infection may be as strategy for the pathogens to modulate the immune responses and eventually leads to the loss of immunological memory [40]. Although the systemic decline of antigen-specific CD4^+^ T cells during *O. tsutsugamushi* infection was not examined in the current study, the systemic apoptosis and lymphopenia of CD4^+^ T cells observed in scrub typhus patients might explain the absence of memory responses observed in vaccine trials and recurrent human infection. In addition, the CD4^+^ T cell lymphopenia observed during the acute phase of scrub typhus may lead to a defect in generating long-lived functional memory CD8^+^ T cells, *i.e.* the helpless CD8^+^ T cell responses, because CD4^+^ T-cell help is critical for generating functionally-competent memory CD8^+^ T-cells, [43], [44].

Interestingly, we also detected a remarkable increase in Ki-67^+^ T cells, indicating that T cells actively proliferate during the acute phase of scrub typhus (Figure 2B). The contradictory finding of increased apoptosis and proliferation of T cells in the peripheral blood of scrub typhus patients prompted us to measure the levels of IL-7 and IL-15, which potently induce the survival and proliferation of T cells [23]. Consistent with another report [45], both of these cytokines were significantly increased in the serum of scrub typhus patients (Figure 2D). Induction of IL-7 and IL-15 may occur as part of a homeostatic response to T-cell depletion and this would account for the recovery of T cells during the convalescent phase. IL-15 is also significantly induced in endothelial cells infected with *O. tsutsugamushi* [46]. Given that CD8^+^ T cells proliferate more efficiently than CD4^+^ T cells in the presence of both cytokines [23], extensive proliferation of CD8^+^ T cells during the convalescent phase (Figure 2B and C) could be explained by an increase in these cytokines. Our current results strongly suggest that T cells, especially CD8^+^ T cells, undergo rapid turnover during *Orientia* infection. This phenomenon has also been observed in other infections [47].

Although Th1-mediated cellular immunity and IFN-γ production by T cells in response to *O. tsutsugamushi* infection is critical for protection, Th1 and Th2 type responses are not clearly polarized in animal infection models [15] or human scrub typhus patients [38]. In this study, we also observed elevated IFN-γ (pg/ml, 24.97 l±24.00 in the patients versus 1.69±4.76 in healthy controls) and IL-10 (pg/ml, 10.75±6.93 *versus* 4.23±7.37) during the acute phase of infection although both cytokines returned to baseline levels during the convalescent phase. The selective reduction of type 1 T cells in the peripheral blood during the acute phase (Figure 3) could be explained by the specific recruitment of these cells to inflamed tissues. Indeed, it was previously shown that IFN-γ-inducible protein 10 (IP-10) and Mig, which bind specifically to CXCR3 expressed on type 1 T cells [24], , are significantly elevated in the plasma of scrub typhus patients [48]. Initial inflammation at the infection site might be initiated by the infiltration of neutrophils [49], followed by the recruitment of monocytes and lymphocytes by several chemokines such as MIP-1 α/β, RANTES, and MCP-1, which are expressed by infected endothelial cells and macrophages upon infection [10], [46], [50]. Systemic elevation of IFN-γ might further enhance the expression of IP-10 and Mig, which lead to local exudation of type 1 T cells to inflamed tissues.

Another interesting finding is the remarkable reduction of CD4^+^ Treg cells in the peripheral blood of scrub typhus patients (Figure 4). A role for Treg cells in *O. tsutsugamushi* infection has never been previously examined; yet evidence from many chronic infectious diseases suggest that Treg cells represent a double-edged sword, limiting both the magnitude of effector responses and the collateral tissue damage caused by vigorous antimicrobial immune responses [26]. Even though the marked reduction of Treg cells in the peripheral blood of scrub typhus patients could be due to the migration and accumulation of suppressive cells to the inflamed tissues as observed in other chronic infections [26], the inverse correlation between a systemic decrease in Treg cells and an increase in proliferating CD8^+^ T cells (Figure 2B and 4B) along with increased CTL activity [48] strongly suggest a functional impairment of Treg cells in scrub typhus patients. Even though further work is required to clearly define the functional status of Treg cells in specific tissues, it could be assumed that the systemic reduction of Treg cells upon *O. tsutsugamushi* infection may significantly contribute to tissue damage by deregulating the proliferation and activation of CD8^+^ T cells. The proliferation of CD8^+^ EM subsets (Figure 5), which may include both activated CTLs and effector memory cells, and a higher frequency of activated CD8^+^ T cells with PD-1^high^ and IL-7Rα^low^ phenotypes in the patients (Figure 6) further support this hypothesis. The significant increase of activated CTLs might play a critical role in anti-*Orientia* immunity as reported in a mouse infection model [51]. One may argue that the proliferation of CD8^+^ EM subsets could be protective and linked to memory response, but this is contradictory to observations drawn from vaccine studies and recurrent human infection. As mentioned above, it is well established that CD8^+^ memory cells developed during the primary infection in the absence of CD4^+^ T cell help are poorly functional although the effector functions of the primary CTL responses are independent of CD4^+^ T cell help [52].

In addition to cytotoxic CD8^+^ T cells, NK cells have also been suggested to play a role in the pathogenesis of scrub typhus [31], [53]. In a mouse model for *Rickettsia* infection, NK cell activity was significantly increased on days 2–6 of infection and depletion of NK cells enhanced the susceptibility of mice to *Rickettsia* infection [54], suggesting NK cells have a significant role in early anti-rickettsial immune responses. In the current study, however, the frequency of total NK cells in scrub typhus patients did not change significantly (Figure S5). Considering that the patients' samples were collected when they were symptomatic (i.e. one to two weeks after initial infection), this may be beyond the period of NK cell activities. Nevertheless, we could detect a substantial decrease in peripheral CD56^bright^ NK cells, a regulatory NK cell subset expressing immunoregulatory cytokines such as IFN-γ and IL-10 depending on stimulation [55], in the patients (Figure S1), suggesting that these cells may migrate to inflamed tissues during the symptomatic period. The local distribution and activation status of NK cell subsets in patients needs to be analyzed further to confirm their role in anti-*Orientia* immunity.

## Supporting Information

Figure S1 **Representative gating strategy to examine the frequencies of CD4^+^ and CD8^+^ T cells in blood lymphocytes.** PBMCs were stained with antibodies against CD3 and CD8 and then analyzed on a flow cytometer. The percentage of each population (right panel) was determined after gating on the lymphocyte population in FSC/SSC plot (left panel).
(TIF)

Figure S2 **Representative gating strategy to examine the frequencies of apoptotic (Annexin V-positive, A) or proliferating (Ki-67-positive, B) cells in CD4^+^ or CD8^+^ T cells.** PBMCs were stained with antibodies against CD3, CD4, and CD8 in addition to annexin V or anti-Ki-67 antibody and then analyzed on a flow cytometer. The percentage of each population was determined after sequential gating on CD4^+^ or CD8^+^ T cells as showed in Figure S1.
(TIF)

Figure S3 **Representative gating strategy to examine the frequencies of type 1 and type 2 T cells based on CXCR3 and CCR4 surface expression in CD4^+^ or CD8^+^ T cells.** PBMCs were stained with antibodies against CD3, CD4, and CD8 in addition to anti-CXCR3 or CCR4 antibodies and then analyzed on a flow cytometer. The percentage of each population was determined after sequential gating on CD4^+^ or CD8^+^ T cells as showed in Figure S1.
(TIF)

Figure S4 **Expression of Foxp3 transcription factor in CD4^+^CD25^++^ T cells.** In order to examine the expression of Foxp3 in CD4^+^ T cells expressing different levels of CD25 on the surface, PBMCs were stained with antibodies against CD3, CD4, and CD25 in addition to anti-Foxp3 antibody and then analyzed on a flow cytometer. Representative histograms showing relative expression of Foxp3 in CD4^+^ T cells differentially expressing CD25 are presented after gating on the each subset as shown in Figure 4A.
(TIF)

Figure S5 **Analysis of natural killer cell subsets in peripheral blood of scrub typhus patients.** PBMCs were stained with antibodies against CD3 and CD56 and then analyzed on a flow cytometer. The frequencies of CD3^−^CD56^+^ (left panel, total), CD3^−^CD56^bright^ (middle panel, regulatory), and CD3^−^CD56^dim^ (right panel, cytotoxic) NK cells were compared with healthy controls (HC, n = 9, open circle) and scrub typhus patients at acute phase (AP, n = 10, gray circle) or convalescent phase (CP, n = 10, black circle). Red bars indicate the mean value and *p* values were obtained using the Mann-Whitney *U* test or Wilcoxon signed-rank test. Statistically significant *p* values (<0.05) are shown in bold.
(TIF)

Table S1 **Demographic data, clinical characteristics of scrub typhus patients.**
(XLS)

Table S2 **Summary of demographic data, clinical characteristics of scrub typhus patients.**
(XLS)

Table S3 **The frequencies and absolute counts of leukocyte subpopulations in the peripheral bloods of scrub typhus patients and healthy controls.**
(XLS)

Table S4 **Summary of absolute counts of leukocyte subpopulations.**
(DOC)
