# Supplementary figures and images for: Phenotypic Characterization of Peripheral T Cells and Their Dynamics in Scrub Typhus Patients
Source: PLoS Negl Trop Dis. 2012 Aug 14;6(8):e1789. doi: 10.1371/journal.pntd.0001789 (PMC3419201; doi:10.1371/journal.pntd.0001789)

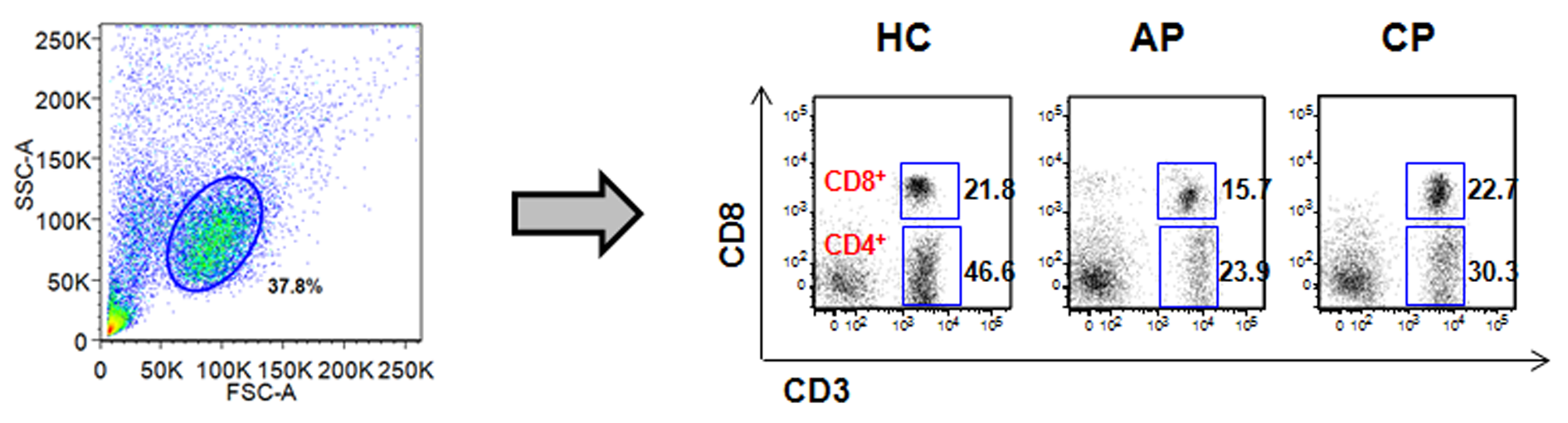

Supplement: Figure S1 — Representative gating strategy to examine the frequencies of CD4+ and CD8+ T cells in blood lymphocytes. PBMCs were stained with antibodies against CD3 and CD8 and then analyzed on a flow cytometer. The percentage of each population (right panel) was determined after gating on the lymphocyte population in FSC/SSC plot (left panel). (TIF) [file pntd.0001789.s001.tif]

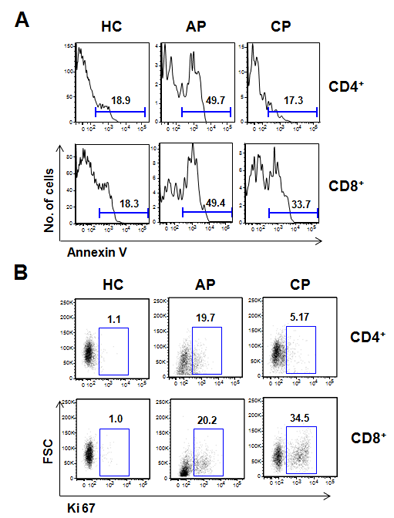

Supplement: Figure S2 — Representative gating strategy to examine the frequencies of apoptotic (Annexin V-positive, A) or proliferating (Ki-67-positive, B) cells in CD4+ or CD8+ T cells. PBMCs were stained with antibodies against CD3, CD4, and CD8 in addition to annexin V or anti-Ki-67 antibody and then analyzed on a flow cytometer. The percentage of each population was determined after sequential gating on CD4+ or CD8+ T cells as showed in Figure S1. (TIF) [file pntd.0001789.s002.tif]

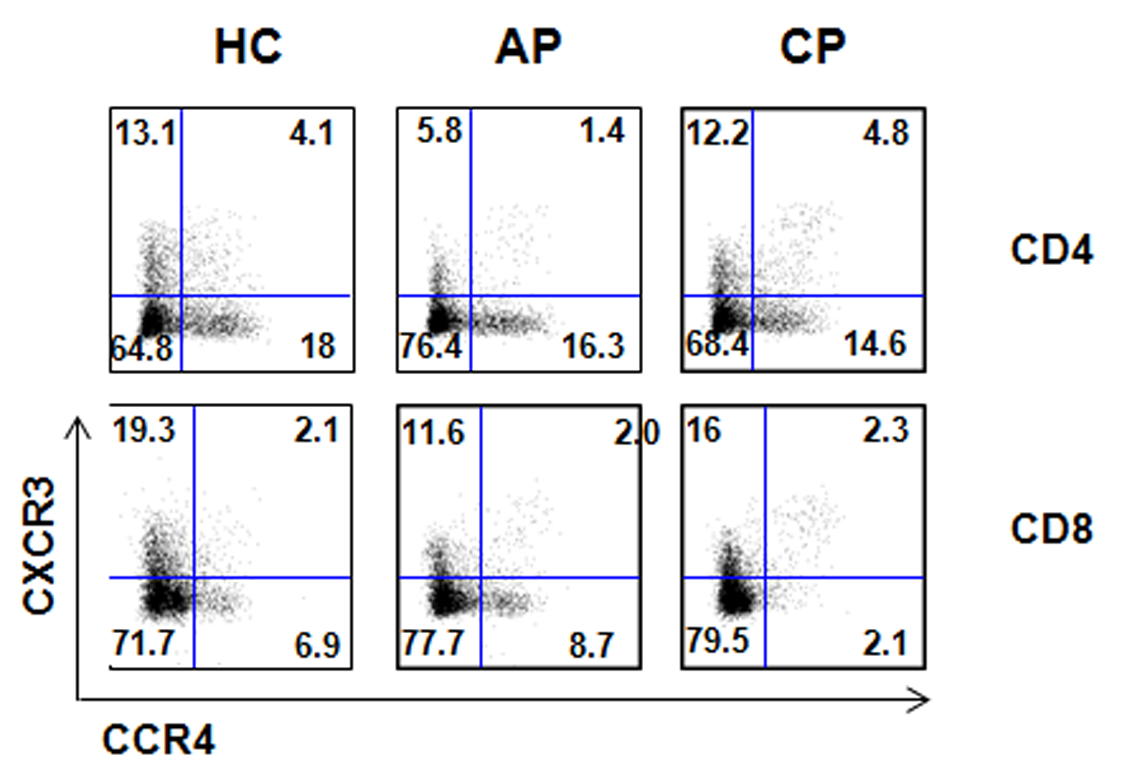

Supplement: Figure S3 — Representative gating strategy to examine the frequencies of type 1 and type 2 T cells based on CXCR3 and CCR4 surface expression in CD4+ or CD8+ T cells. PBMCs were stained with antibodies against CD3, CD4, and CD8 in addition to anti-CXCR3 or CCR4 antibodies and then analyzed on a flow cytometer. The percentage of each population was determined after sequential gating on CD4+ or CD8+ T cells as showed in Figure S1. (TIF) [file pntd.0001789.s003.tif]

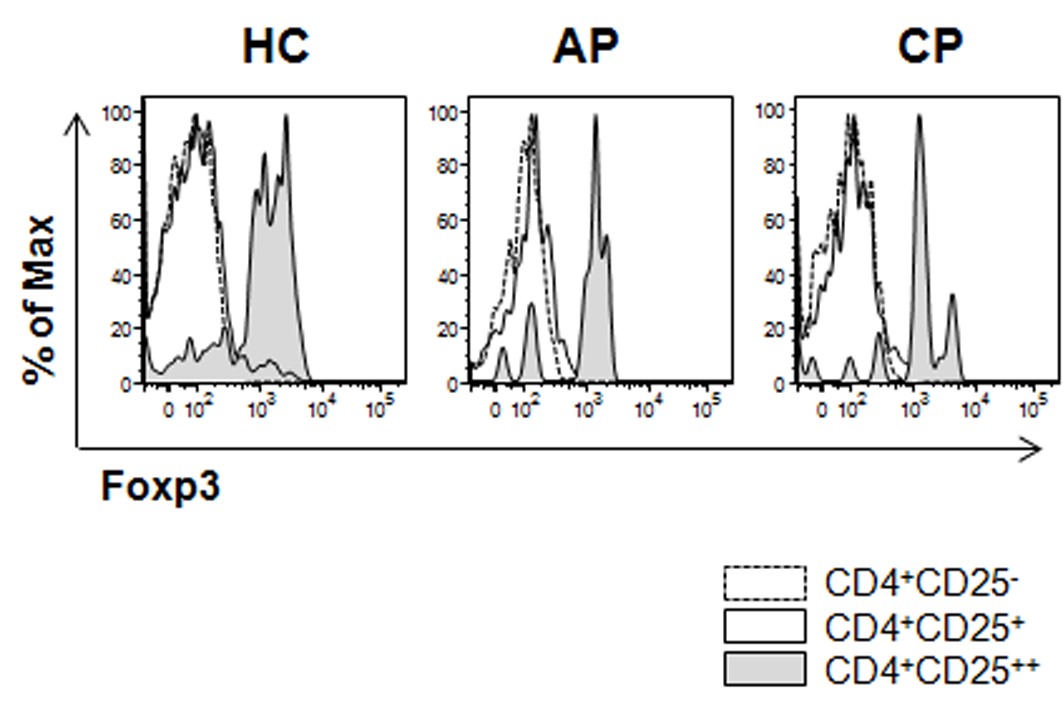

Supplement: Figure S4 — Expression of Foxp3 transcription factor in CD4+CD25++ T cells. In order to examine the expression of Foxp3 in CD4+ T cells expressing different levels of CD25 on the surface, PBMCs were stained with antibodies against CD3, CD4, and CD25 in addition to anti-Foxp3 antibody and then analyzed on a flow cytometer. Representative histograms showing relative expression of Foxp3 in CD4+ T cells differentially expressing CD25 are presented after gating on the each subset as shown in Figure 4A. (TIF) [file pntd.0001789.s004.tif]

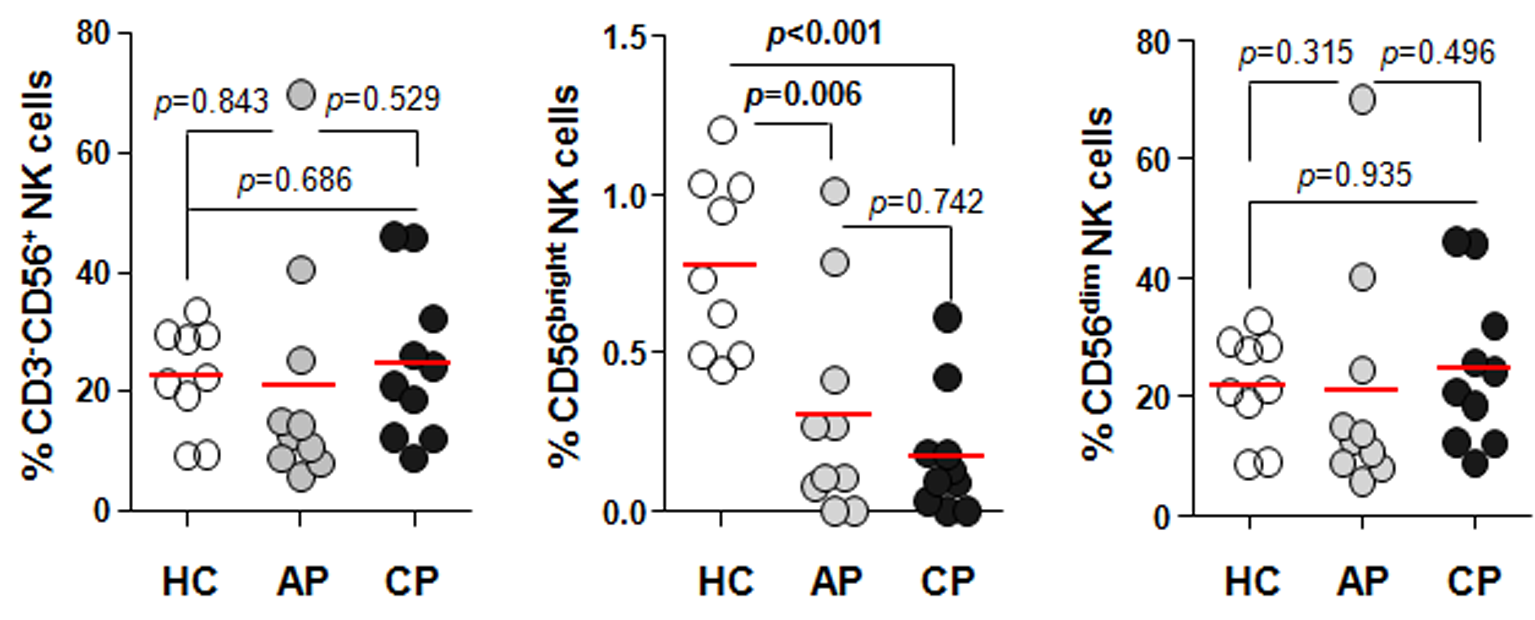

Supplement: Figure S5 — Analysis of natural killer cell subsets in peripheral blood of scrub typhus patients. PBMCs were stained with antibodies against CD3 and CD56 and then analyzed on a flow cytometer. The frequencies of CD3−CD56+ (left panel, total), CD3−CD56bright (middle panel, regulatory), and CD3−CD56dim (right panel, cytotoxic) NK cells were compared with healthy controls (HC, n = 9, open circle) and scrub typhus patients at acute phase (AP, n = 10, gray circle) or convalescent phase (CP, n = 10, black circle). Red bars indicate the mean value and p values were obtained using the Mann-Whitney U test or Wilcoxon signed-rank test. Statistically significant p values (<0.05) are shown in bold. (TIF) [file pntd.0001789.s005.tif]
