# Supplementary material for: Phenotypic Characterization of Peripheral T Cells and Their Dynamics in Scrub Typhus Patients
Source: PLoS Negl Trop Dis. 2012 Aug 14;6(8):e1789. doi: 10.1371/journal.pntd.0001789 (PMC3419201; doi:10.1371/journal.pntd.0001789)
Supplement: Table S4 — Summary of absolute counts of leukocyte subpopulations. (DOC) [file pntd.0001789.s009.doc]

Supplementary Table 4.

|  | HC | AP | CP |
| --- | --- | --- | --- |
| WBC | 5794.4±742.2 | 7175.3±2296.4 | 7012.4±1844.5 |
| Neutrophils | 2810.3±465.7 | 4445.6±1882.0 | 3034.2±1643.6 |
| Monocytes | 416.9±128.4 | 505.3±322.4 | 614.7±195.5 |
| Lymphocytes | 2371.7±506.2 | 1814.6±1205.6 | 3092.3±1025.9 |
| CD4^+^ T cells | 1072.8±382.4 | 498.5±387.9 | 975.1±395.2 |
| CD4^+^AnnexinV^+^ (apoptotic)  CD4^+^Ki-67^+^ (proliferating)  CD4^+^CD25^++^ T cells | 100.8±40.7  3.4±0.9  23.6±6.2 | 94.0±74.7  15.4±12.6  1.7±1.4 | 92.4±42.1  5.0±2.9  8.8±9.7 |
| CD4^+^Foxp3^+^ T cells | 39.8±11.6 | 3.3±1.8 | 22.8±22.1 |
| CD8^+^ T cells  CD8^+^AnnexinV^+^ (apoptotic)  CD8^+^Ki-67^+^ (proliferating) | 421.8±276.7  79.8±29.1  5.6±1.9 | 329.4±349.5  352.0±285.6  250.2±300.8 | 681.8±395.4  179.3±89.7  226.9±209.8 |
| CD8^+^IL7Ra^low^ | 251.1±256.0 | 305.0±300.7 | 594.2±421.1 |
| CD8^+^PD1^high^ | 38.0±27.7 | 94.7±85.3 | 101.2±62.6 |

* absolute counts of leukocyte subpopulations (cells/μl, mean±SD)
